# Acute ingestion of acetaminophen improves cognitive and repeated high intensity short-term maximal performance in well-trained female athletes: a randomized placebo-controlled trial

**DOI:** 10.1007/s00421-024-05534-y

**Published:** 2024-06-28

**Authors:** Sahar BenSalem, Atef Salem, Omar Boukhris, Morteza Taheri, Achraf Ammar, Nizar Souissi, Jorden M. Glenn, Khaled Trabelsi, Hamdi Chtourou

**Affiliations:** 1 High Institute of Sport and Physical Education of Sfax, University of Sfax, Sfax, Tunisia; 2 Research Laboratory: Education, Motricity, Sport and Health, EM2S, LR19JS01, High Institute of Sport and Physical Education of Sfax, University of Sfax, Sfax, Tunisia; 3 Department of Training and Movement Science, Institute of Sport Science, Johannes Gutenberg-University Mainz, 55122 Mainz, Germany; 4 Research Unit, Physical Activity, Sport, and Health, UR18JS01, National Observatory of Sport, 1003 Tunis, Tunisia; 5 SIESTA Research Group, School of Allied Health, Human Services and Sport, La Trobe University, Melbourne, VIC 3086 Australia; 6 Sport, Performance, and Nutrition Research Group, School of Allied Health, Human Services and Sport, La Trobe University, Melbourne, VIC 3086 Australia; 7 Department of Behavioral and Cognitive Sciences in Sport, University of Tehran, Tehran, Iran; 8 Department of Health, Exercise Science Research Center Human Performance and Recreation, University of Arkansas, Fayetteville, AR 72701 USA; 9 Research Laboratory, Molecular Bases of Human Pathology, LR19ES13, Faculty of Medicine of Sfax, University of Sfax, Sfax, Tunisia

**Keywords:** Paracetamol, Repeated sprints, Mood, Attention, Fatigue

## Abstract

This study examined the effect of acute acetaminophen (ACTP) ingestion on physical performance during the 5 m shuttle run test (5mSRT), attention, mood states, and the perception of perceived exertion (RPE), pain (PP), recovery (PRS), and delayed onset of muscle soreness (DOMS) in well-trained female athletes. In a randomized, placebo-controlled, double-blind, crossover trial, fifteen well-trained female athletes (age 21 ± 2 years, height 165 ± 6 cm, body mass 62 ± 5 kg) swallowed either 1.5 g of ACTP or 1.5 g of placebo. The profile of mood states (POMS) and digit cancellation (DCT) were assessed 45 min postingestion, and 5mSRT was performed 60 min postingestion. The RPE and PP were determined immediately after each 30-s repetition of the 5mSRT, and the PRS and DOMS were recorded at 5 min and 24 h post-5mSRT. For the 5mSRT, ACTP ingestion improved the greatest distance (+ 10.88%, *p* < 0.001), total distance (+ 11.33%, *p* = 0.0007) and fatigue index (+ 21.43%, *p* = 0.0003) compared to PLA. Likewise, the DCT score was better on the ACTP (*p* = 0.0007) than on the PLA. RPE, PP, PRS, and DOMS scores were improved after ACTP ingestion (*p* < 0.01 for all comparisons) compared to PLA. POMS scores were enhanced with ACTP ingestion compared to PLA (*p* < 0.01). In conclusion, this study indicates that acute acetaminophen ingestion can improve repeated high intensity short-term maximal performance, attention, mood states, and perceptions of exertion, pain, recovery, and muscle soreness in well-trained female athletes, suggesting potential benefits for their overall athletic performance and mood state.

## Introduction

Sports-based activities depend on both physiological and psychological (i.e., emotional and cognitive) requirements. Physical performance has a dynamic relationship with psychological state (Beal et al. 2005). However, athletes face difficult moments (e.g., precompetitive negative emotions, professional constraints) and may experience disturbances in their psychological states during training and/or competition (Merino Fernández et al. 2019). These disturbances can lead to performance decrements (Carrier and Debois 2003; Gaudreau et al. 2010). Women generally tend to report a higher disruption of temporary emotional state than men (Domes et al. 2010; Damien and Mendrek 2017). Additionally, it is recognized that psychological disturbances can influence the perception and tolerance of pain in both women and men (Jones et al. 2003; Tang and Gibson 2005).

Different strategies must be evaluated to effectively reduce underlying mood disturbances, cognitive performance, and pain perception. For example, previous studies report that acetaminophen (ACTP, paracetamol) has the potential to be a pharmacological strategy for pain relief (Mauger et al. 2010), improving physical (Grgic 2022) and cognitive performance (Pickering et al. 2016), as well as emotional state (DeWall et al. 2010; Manna and Umathe 2015). ACTP is an over-the-counter pain reliever and one of the most widely used medications for pain and fever reduction (Sood et al. 2013). Additionally, ACTP has multiple effects on the central nervous system (Umathe et al. 2009; Manna and Umathe 2015). Although a century has passed since its discovery, the full mechanism of action of ACTP remains unknown (Tanner et al. 2010). However, the main mechanism of action to reduce pain is the inhibition of cyclooxygenase (i.e., the enzyme responsible for the production of prostaglandins from arachidonic acid) (Anderson 2008), modulating afferent and efferent pain pathways (Andersson et al. 2011). ACTP is similar to nonsteroidal anti-inflammatory drugs (NSAIDs), but due to its limited anti-inflammatory action, it is not classified as an NSAID (Graham et al. 2013). Furthermore, ACTP does not directly affect cyclooxygenase (COX) 1 and 2 enzymes; however, it can exert its effects on an enzyme known as COX-3, which is generated through the splicing process of COX-1 (Chandrasekharan et al. 2002; Przybyła et al. 2021). This mechanism leads to the absence of gastrointestinal lesions, adverse cardiorenal effects, and antiplatelet effects (Bertolini et al. 2006). ACTP has a central mechanism of action that involves several neurotransmitters and serotonergic, cannabinoid, opioid, and vanilloid receptors (Sandrini et al. 2007; Ohashi and Kohno 2020). Additionally, ACTP acts on cannabinoid type 1 (CB1) receptors and calcium channels, such as Ca(v), and regulates descending serotonergic pathways and a number of other factors, including A1 receptor potential members of the transient receptor cation channel (Ohashi and Kohno 2020; Przybyła et al. 2021).

The cognitive and emotional variations with ACTP have been examined in animals, and reports suggest that there is an anxiolytic effect (Umathe et al. 2009), which is associated with improved cognitive function (Ishida et al. 2007). The utilization of the cannabinoid mechanism and the augmentation of the antidepressant effect are two key factors through which a small amount of ACTP can facilitate effective management of depression (Manna and Umathe 2015). Preclinical investigations conducted on animal models have demonstrated that administering therapeutic doses of ACTP may enhance cognitive abilities, potentially attributed to its impact on the central serotonergic system. This leads to an augmented release of serotonin and norepinephrine within the brain, thereby potentially improving cognitive performance (Maharaj et al. 2004; Blecharz-Klin et al. 2013). Overall, ACTP seems to enhance cognitive performance, especially attention, and has been shown to do so during the decision-making test and spatial memory, with an acute dose of 2 g in healthy men (Pickering et al. 2016). In addition, using functional magnetic resonance imaging to measure brain activity in healthy participants, it was found that administration of 1 g of ACTP reduced negative emotions in certain brain regions (anterior insula, dorsal anterior cingulate cortex) (DeWall et al. 2010). Furthermore, it has been shown that this ACTP is the most widely used by athletes (Lundberg and Howatson 2018). Traditionally, ACTP has been used in athletes to alleviate the pain of physical exercise (Esh et al. 2017). However, it is also considered an ergogenic aid, which can be misused to enhance sports performance. While the benefits of this drug appeared when administered at therapeutic doses (Prior et al. 2012), ACTP exhibits minimal anti-inflammatory activity, functioning merely as a modest inhibitor of prostaglandin production (Botting 2000), and its analgesic properties are not altered during exercise (Sawrymowicz 1997).

Most studies have focused on the influence of the ACTP on endurance performance (Grgic and Mikulic 2021), and numerous studies have examined the effects of the ACTP on the performance of short-term repeated sprints interspersed with 2–4 min of recovery (Foster et al. 2014, Delextrat et al. 2015). The ability to repeat high-intensity efforts is essential for performance in many sporting specialties (Fernandez-Fernandez et al. 2012; Eryılmaz et al. 2019). Additionally, one of the most important factors in the success of high-intensity exercise is the ability of the athlete to tolerate pain (O’connor 1992).

Indeed, the acute ingestion of 1.5 g ACTP has been shown to improve repeated sprint performance in the Wingate test (8 × 30 s interspersed with 2 min rest) (Foster et al. 2014, Delextrat et al. 2015).

Most studies performing short-term repeated sprint tests after ACTP ingestion were performed on a bicycle ergometer or isokinetic dynamometer for a single member (Foster et al. 2014, Delextrat et al. 2015, Morgan et al. 2018); only one study used a running-based protocol on a treadmill (Park et al. 2016). Therefore, to better understand the influence of ACTP on maximum short-term performance, a field test, such as the 5 m shuttle run test (5mSRT), which involves more muscle activation compared to efforts on a treadmill (Baur et al. 2007; Sedıghı et al. 2019) or bicycle ergometers, is warranted.

The 5mSRT, adopted by the Welsh Rugby Union and modified by the Sports Science Institute of South Africa (Boddington et al. 2001), is one of the most widely used short-duration, high-intensity repeated sprint tests to determine an athlete’s fitness (Pendleton 1997; Boddington et al. 2004). The test consists of maximal shuttle sprints of 6 × 30 s with 35 s recovery in between. In this test, athletes run the greatest possible distance for 30 s, going back and forth over 5 m, then 10 m, then 15 m, then 20 m and so on. In addition, during the 5mSRT, both aerobic and anaerobic metabolisms could be solicited since its total duration is around 6 min (i.e. 6 sprints of 30 s with 35 s rest between sprints) generating high levels of fatigue, biomarkers of muscle damage and inflammation, as well as perceived exertion (RPE), delayed onset muscle soreness (DOMS) and reduced perception of recovery (PRS) (Boukhris et al. 2020).

It is well documented that women are more sensitive to pain than men (Naugle et al. 2014; Templeton 2020) and may also react differently to ACTP (Bartley and Fillingim 2013). Traditionally, there is a lack of participation of women in sports medicine research (Costello et al. 2014), and only one study has examined the effects of ACTP on physical performance in trained females (Delextrat et al. 2015).

To the author’s knowledge, no study has examined the effects of acute ingestion of ACTP on attention and mood states in well-trained female athletes. Thus, this study investigated the influence of an acute ACTP on physical performance during the 5mSRT, rating of RPE, pain perception (PP), DOMS, PRS, attention, and mood states in well-trained female athletes. We hypothesized that ACTP ingestion would have a positive performance effect during the 5mSRT, attention, mood state and PRS as well as reduce PP, DOMS and RPE in female athletes.

## Materials and methods

### Participants

The minimum needed sample size was calculated using G*power software (version 3.1.9.6; Kiel University, Kiel, Germany) (Faul et al. 2007). We set the values of α and power (1−β) at 0.05 and 0.95, respectively. Based on Delextrat et al. (2015)’s physical performance results and discussions between the authors, the effect size was estimated to be *d* = 1.1; the needed sample size was thirteen. Fifteen well-trained female athletes (age = 21 ± 2 years, height = 165 ± 6 cm, weight = 62 ± 5 kg) voluntarily participated in the study. The participants practiced combat sports (English boxing) in local clubs and regularly trained 5 days a week for an average of 2 h of session; each had at least 4 years of sporting experience and participated in at least 4 regional/national competitions per year. Prior to starting the study, volunteers were asked to provide written informed consent and complete a Physical Activity Readiness Questionnaire and an ACTP risk assessment questionnaire (Mauger 2009). This study was carried out in accordance with the Declaration of Helsinki, and the protocol has been fully validated by the Ethical Committee for the Protection of Southern Persons (CPP SUD No. 0418/2022). Participants were in good health, did not have kidney or liver disease, did not use anti-inflammatory and analgesic substances (by prescription and over the counter), and did not consume alcohol or smoke cigarettes. They were also needed to not take birth control pills, as taking combined oral contraceptive pills can help stabilize hormonal fluctuations (Vincent and Tracey 2008) and may decrease the perception of experimental pain in women (Dao 2012).

### Experimental design

This study utilized a randomized, placebo-controlled, double-blind, crossover design. The 5mSRT was carried out on a tarmac running track. During the experimental period, temperature, humidity and wind ranged from 20 to 22 °C, from 52 to 60% and from 2.5 to 4.16 m. s^−1^, respectively.

Prior to each visit, participants were asked to abstain from any strenuous exercise for 24 h and caffeine for 12 h before the experiment. They were also asked to refrain from taking analgesics or any form of anti-inflammatory medication during the experiment. After a familiarization session with the experimental procedure (i.e. with the 5mSRT and the questionnaires), participants were asked to maintain their diet and arrive in a state of perfect rest and hydration to perform two separate sessions for a minimum of 72 h where this was verbally confirmed before the start of the trial.

Participants were assigned to a placebo (PLA; Maltodextrin) or a 1.5 g of ACTP, with the supplements enclosed in three small gelatin-coated capsules (3 × 500 mg). The study used a crossover design, where participants switched to the alternate supplementation condition after the initial phase.

For the first group of seven participants:

- Four participants receive PLA first, followed by ACTP.
- Three participants receive ACTP first, followed by PLA.

For the second group of eight participants:

o Four participants receive ACTP first, followed by PLA.
o Four participants receive PLA first, followed by ACTP.

The Profile of Mood Profile (POMS) questionnaire and the digit cancellation test were assessed at 45 min post ingestion. Likewise, the 5mSRT was performed at 60 min post ingestion. This period was chosen because maximum plasma concentrations of ACTP are observed at 30–60 min post ingestion (Anderson 2008). Acute administration of 1.5 g ACTP had a longer plasma half-life and showed no signs of impaired glutathione conjugation or hepatotoxicity in healthy individuals or those with chronic liver disease (Forrest et al. 1979; Alchin et al. 2022). Furthermore, the ergogenic effect of ACTP is most frequently observed when 1.5 g is ingested 30–60 min before physical exercise (Grgic 2022).

To mitigate the effects of diurnal variations, the tests were executed at the same time of day (Hayes et al. 2010), at 17 h 00, for all participants.

During the execution of the 5mSRT test, participants responded to RPE and PP scales immediately after each sprint during recovery periods (i.e. between repetitions). Additionally, participants responded to the PRS scale 5 min post-5mSRT. The DOMS scale was determined 24 h after the end of the 5mSRT. Standardized verbal explanations of the appropriate uses of all questionnaires were provided prior to testing.

### Profile of mood states (POMS)

Subjective mood status was assessed using the French version of the POMS questionnaire (Cayrou et al. 2003). Responses to each element range from 0 to 4, with the highest scores indicating a more negative mood (0 indicates “Not at all” and 4 indicates “Extremely”). The POMS assesses mood status (McNair et al. 1971); it contains 65 adjectives and assesses six mood factors: depression, fatigue, tension, anger, vigor, and confusion. Total mood disturbance (TMD) can be calculated by adding the scores for tension, depression, anger, fatigue and confusion and then subtracting the score for vigor.

### The digit-cancellation test (DCT)

As described by Hatta et al. (2012), the DCT consists of deleting target numbers (i.e., numbers composed of three grouped digits) and circulating them as much as possible in a limited time (1 min), working line by line, from left to right, leaving aside all the other numbers that were not composed of three digits. The test paper contained 600 signs divided into 36 lines. The sum of correct responses was recorded for analysis.

### 5-m shuttle run test (5mSRT)

This test consisted of performing 6 repetitions of 30-s shuttle sprints interspersed with a 35-s recovery period. Participants sprinted a maximum distance, back and forth 5 m, then 10 m, then 15 m, then 20 m, etc., for 30 s. After each 30-s repetition, a 35-s recovery was allowed. During the recovery phase, participants returned to the starting position for the next repetition (Boddington et al. 2001).

Depending on the distance covered during each repetition, the following parameters were calculated as used by Boukhris et al. (2022):

- Greatest distance (GD) (m) = the greatest distance traveled during a 30 s sprint.
- Total distance (TD) (m) = the total distance traveled during the six 30 s shuttles.
- The fatigue index (FI) was calculated as follows:
  $$\text{FI} (\text{\%})=\begin{array}{c} \\ \frac{\left[\frac{\left(\text{shuttle }1+\text{ shuttle }2\right) }{2}-\frac{(\text{shuttle }5+\text{ shuttle }6 )}{2}\right]}{\frac{\left(\text{shuttle }1+\text{ shuttle }2\right)}{2}}\end{array} \times 100$$

### Pain perception (PP)

After each 30-s repetition, participants selected a score on a 10-point scale accompanied by verbal descriptions to assess perceived pain between conditions. The high intraclass correlations (*r* = 0.88–0.98) indicate that this scale is a reliable measure of pain during effort (Cook et al. 1997).

### Rating of perceived exertion (RPE)

After each 5mSRT repetition, participants completed their subjective RPE score from 0 (very, very light) to 10 (very, very hard). The higher the RPE score is, the greater the degree of effort. This scale is a good indicator of physical exertion, strongly correlated with several physiological measures of exertion, and has good psychometric properties (Haddad et al. 2013, Boukhris et al. 2020). The following formula was applied to obtain the average RPE score in the 5mSRT test:

$$\text{RPE }\left(\text{AU}\right)= \frac{\text{Sum of RPE scores for all repetitions}}{\text{Number of repetitions}}$$

### Delayed onset muscle soreness (DOMS)

Delayed onset of muscle soreness (DOMS) was assessed at 24 h after the 5mSRT using a numerical scale ranging from 0 to 10 (Hawker et al. 2011). The values on the scale ranged from 0 “no pain” to 10 “very, very painful”.

### Perceived recovery status (PRS)

PRS was assessed 5 min after the 5mSRT using an 11-point scale ranging from “0” to “10;” 0–2 represents “recovery is very low and performance is expected to decrease,” 4–6 means “recovery is low to moderate and performance is expected to be similarly good,” and 8–10 means “perceived high recovery and performance is expected to improve” (Laurent et al. 2011).

### Statistical analysis

**Fig. 1:**
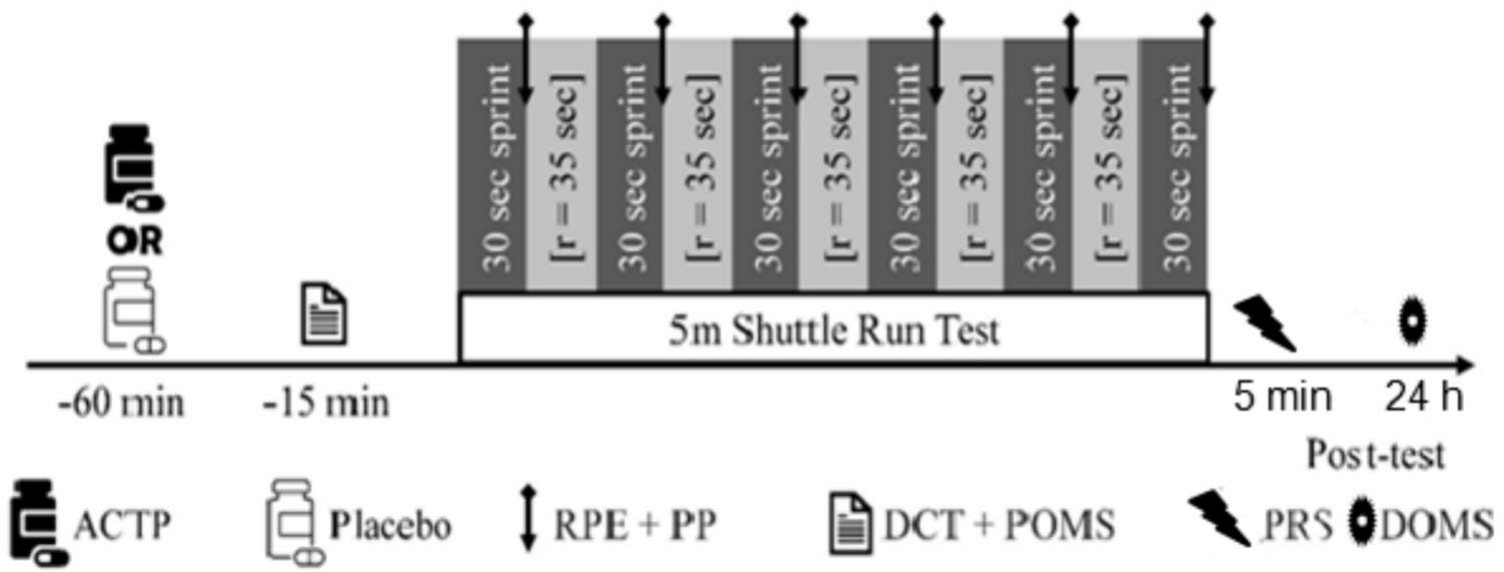
Schematic representation of the experimental protocol. *ACTP* Acetaminophen, *RPE* Rating of perceived exertion, *PP* Pain perception, *DCT* Digit cancelation test, *POMS* Profile of mood states, *PRS* Perceived recovery status, *DOMS* Delayed onset of muscle soreness, *r* Recovery

Values are expressed as the mean ± standard deviation (SD). Statistical analyses were performed using STATISTICA (StatSoft, France, version 10). The normality of the distributions was confirmed by the Shapiro‒Wilk test. The dependent t test was performed for tension, depression, POMS total score, GD, FI, PP and RPE. However, normality was not confirmed for attention, vigor, anger, DOMS, PRS, and TD; therefore, the Wilcoxon test was used. Standardized effect size (Cohen’s d) analysis was used to interpret the magnitude of differences between variables. Significance was accepted for all analyses at the level of *p* < 0.05. When the STATISTICA output demonstrated significance levels of *p* = 0.0000, these were corrected to *p* < 0.0001 (Fig. 1).

To determine the percentage of gain or decrease with ACTP compared to PLA for all parameter, Δ was calculated as follows:

$$\Delta \text{\%}=\left[\frac{\left(\text{Higher value}-\text{ lower value }\right)}{\text{Higher value}}\right] \times 100$$

## Results

### 5-m shuttle run test

**Fig. 2:**
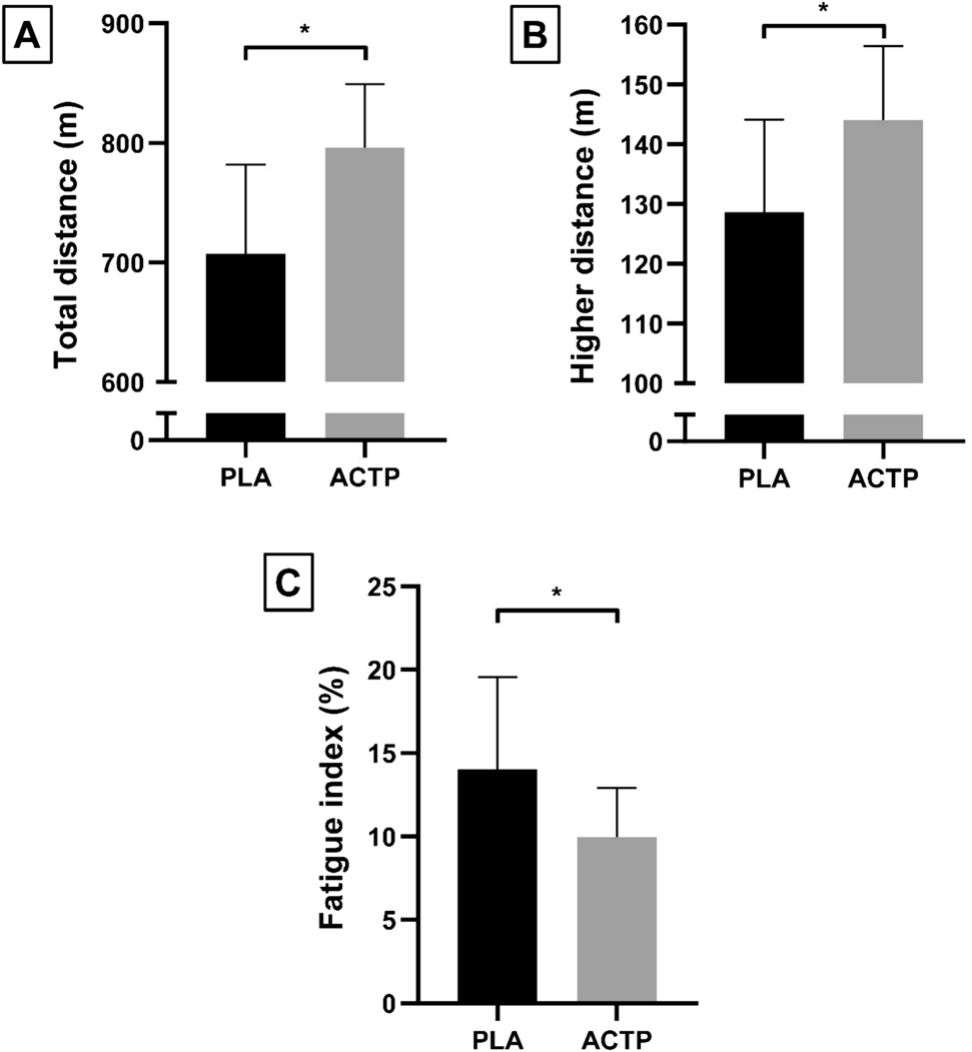
The total distance (**A**), greatest distance (**B**), and fatigue index (**C**) recorded after ingestion of placebo (PLA) and acetaminophen (ACTP). *(*p* < 0.001): significant difference compared to PLA

The TD, GD, and FI during the 5mSRT are presented in Fig. 2. Statistical analysis revealed that TD (+ 11.33%) and GD (+ 10.83%) were significantly higher in the ACTP condition than in the PLA condition (*Z* = 3.41, *p* = 0.0007, *d* = 1.38 and *t* = 8.29, *p* < 0.0001, *d* = 1.1, respectively). Additionally, FI was significantly lower by 21.44% in the ACTP condition than in the PLA condition (*t* = 4.77, *p* = 0.0003, *d* = 0.91).

### The digit-cancellation test (DCT)

Statistical analysis showed that DCT scores were higher after ACTP ingestion than after PLA ingestion (*Z* = 3.4, *p* = 0.0006, *d* = 1.09) (Table 1).

**Table 1:** Values are expressed as the mean ± SD

	PLA	ACTP	∆%	*t/Z*	*p* value	Cohen’s d
DCT (AU)	80.4 ± 10.08	89.7 ± 6.98	10.54	*Z* = 3.41	0.0007	1.09
RPE (AU)	5.6 ± 0.5	3.2 ± 0.6	− 40.33	*t* = 10.56	< 0.0001	3.54
PP (AU)	5.8 ± 0.7	3 ± 0.5	− 42.55	*t* = 12.66	< 0.0001	4.14
PRS (AU)	4 ± 0.9	6.9 ± 0.7	40.46	*Z* = 3.41	0.0007	3.40
DOMS (AU)	6 ± 1.1	4 ± 0.6	− 31.76	*Z* = 3.29	0.001	2.3

Digit cancelation test (DCT), delayed onset muscle soreness (DOMS), perceived recovery status scale (PRS), rating of perceived exertion scale (RPE), and perceived pain (PP) recorded after the consumption of the ACTP and the PLA

*AU* arbitrary units

### Rating of perceived exertion scale (RPE)

Statistical analysis showed that RPE values were lower after ACTP ingestion than after PLA ingestion (*t* = 10.56, *p* = ** < **0.0001, *d* = 3.54) (Table 1).

### Rating of perceived pain (PP)

Statistical analysis showed that PP values were lower after ACTP ingestion than after PLA ingestion (*t* = 12.66, *p* = ** < **0.0001, *d* = 4.14) (Table 1).

### Perceived recovery status (PRS)

Statistical analysis showed that PRS values were higher after ACTP than after PLA (*Z* = 3.41, *p* = 0.0006, *d* = 3.40) (Table 1).

### Delayed onset muscle soreness (DOMS)

Statistical analysis showed that DOMS values were lower after ACTP than after PLA (*Z* = 3.29, *p* = 0.001, *d* = 2.3) (Table 1).

### Profile of mood states (POMS)

Analyses revealed a significant effect of ACTP ingestion compared to PLA for anxiety, anger, depression, fatigue, vigor, confusion, and TMD score. Anxiety (*t* = 13.31, *p* = ** < **0.0001, *d* = 2.29), depression (*t* = 20.14, *p* = ** < **0.0001, *d* = 0.86), anger (*Z* = 3.41, *p* = 0.0007, *d* = 0.97), fatigue (*Z* = 3.41, *p* = 0.0007, *d* = 0.95), confusion (*Z* = 3.3, *p* = 0.001, *d* = 0.77) and TMD (*t* = 19.63, *p* = ** < **0.0001, *d* = 1.83) were reduced with the ACTP compared to the PLA. However, vigor (*Z* = 3.41, *p* = 0.0007, *d* = 1.16) was increased with ACTP compared to PLA (Table 2).

**Table 2:** Values of the profile of mood states scores recorded after ingestion of the ACTP and the PLA

	PLA	ACTP	*t/Z*	*p* value	Cohen’s d
Anxiety (AU)	9.7 ± 3	4 ± 1.9	*t* = 13.31	< 0.0001	2.29
Depression (AU)	10 ± 4	6 ± 4	*t* = 20.14	< 0.0001	0.86
Anger (AU)	10.5 ± 6.1	5.6 ± 4.5	*Z* = 3.41	0.0007	0.97
Vigor (AU)	16.4 ± 4.8	21.6 ± 3.9	*Z* = 3.41	0.0007	1.16
Fatigue (AU)	7 ± 4	3 ± 3	*Z* = 3.41	0.0007	0.95
Confusion (AU)	8 ± 3.4	4.5 ± 2.2	*Z* = 3.3	0.001	0.77
TMD (AU)	29 ± 16	7.9 ± 13	*t* = 19.63	< 0.0001	1.83

*AU* arbitrary units, *TMD* total mood disturbance

## Discussion

The present study investigated the influence of acute ACTP ingestion on 5mSRT performance, RPE, PP, DOMS, PRS, attention, and mood states in well-trained female athletes. Although some previous studies have investigated physical performance, to the authors’ knowledge, this is the first study examining the effect of ACTP ingestion on cognitive performance and mood states in trained females. Our findings show that acute ACTP ingestion effectively enhanced physical performance during the 5mSRT, attention and mood states.

For cognitive performance and emotional state, our results are consistent with previous reports showing that a nontoxic dose can improve cognitive performance (Blecharz-Klin et al. 2013; Pickering et al. 2016) and emotional state (DeWall et al. 2010; Manna and Umathe 2015). Moreover, preclinical studies report improvements in cognitive performance after a low dose of ACTP (Ishida et al. 2007; Blecharz-Klin et al. 2013). A clinical study indicated that acute ingestion of a nontoxic dose of ACTP improves attention, with better memory acquisition and a tendency to improve problem solving during cognitive tests (Pickering et al. 2016). ACTP may have a pharmacological mechanism that reacts with a variety of physiological pathways, such as the serotonin (5-HT) and endocannabinoid systems (Pickering et al. 2006; Sandrini et al. 2007). Interestingly, the ACTP metabolite *N*-arachidonoyl-phenolamine induces CB1 receptor activation and acts as a full agonist of the transient receptor potential vanilloid type 1 (TRPV1) receptor (Rawls et al. 2006). Activation of the TRPV1 receptor has been shown to produce antidepressant and anxiolytic efficacy by modulating serotonergic transmission (Manna and Umathe 2015). Moreover, the endocannabinoid system acts on cognitive function, as it improves attention and mood states via CB1 receptors localized to noradrenergic axon terminals, with norepinephrine release and stimulation of the α2 adrenergic receptor (Mendiguren and Pineda 2004; Cathel et al. 2014). Additionally, it has been reported that ACTP increases 5-HT and noradrenaline levels in the brains of rats (Maharaj et al. 2004; Blecharz-Klin et al. 2013).

Regarding emotional state, the ACTP may regulate emotions by decreasing daily feelings of psychological suffering (DeWall et al. 2010). In the present study, mood states estimated by the POMS (i.e., anxiety, depression, anger, vigor, fatigue, confusion, and TMD) were improved by the ACTP. The anxiolytic-type effect of ACTP could be a main feature to improve mood states (Umathe et al. 2009; Viberg et al. 2014). Additionally, a single dose of ACTP (which does not reach the threshold of fluoxetine*,* a selective serotonin reuptake inhibitor), increases the antidepressant-type effect and may provide better management of depression (Manna and Umathe 2015). Furthermore, 5-HT is a key neurotransmitter involved in mood state regulation (Redelinghuys 2020) and modulates emotion (Meltzer 1990). A significant observation arises from the gender context, where women tend to face a heightened susceptibility to mood and mental disorders (Organization 2022), attributed in part to a 50% reduction in 5-HT synthesis within the central nervous system compared to men (Oh et al. 2023), coupled with an elevated abundance of serotonin transporters (Gressier et al. 2016). Therefore, a therapeutic dose of ACTP can modulate the release/recapture processes of 5-HT (Blecharz-Klin et al. 2013); the antidepressant effect of ACTP may also rebalance serotonin levels (Manna and Umathe 2015). This may explain the improvements in cognitive performance and mood states in our study.

In contrast, our results revealed a significant improvement in physical performance during the 5mSRT (i.e., + 11.33% for TD, + 10.83% for GD and 21.44% for FI). Our result was consistent with studies investigating the effect of ACTP ingestion on performance in repeated sprints (Foster et al. 2014, Delextrat et al. 2015, Morgan et al. 2018).

Foster et al. (2014) used eight bouts of the Wingate test (30 s of all-out cycling) interspersed with 2 min of rest, reporting that ACTP ingestion increased by 5% during the Wingate’s 8 bouts and by 10–11% only during the last 3 bouts (Six to eight bouts) in physically active male participants. Delextrat et al. (2015) used the same Wingate exercise protocol (i.e. 8 × 30 s interspersed with 2 min recovery) and the same ACTP intake used by Foster et al. (2014), but included physically active women, they found an increase in mean power (6%) and more specifically, higher mean power values were observed during the second, third and fifth bouts (11–13%). Previous studies (Foster et al. 2014; Delextrat et al. 2015; Park et al. 2016), reported that the ACTP during the first sprint was not ergogenic. Therefore, it seems that acute ingestion of 1.5 g ACTP is effective in attenuating power loss during repeated sprints.

Indeed, ACTP could improve the reactivity and excitability of the corticospinal tract (Mauger and Hopker 2013). Furthermore, an acute ACTP ingestion can improve muscle activation during maximal intermittent exercise (Morgan et al. 2018). Given that intense repeated sprint exercise has been associated with a decrease in cortical excitability (Pearcey et al. 2016), and that the 5mSRT being a maximal exercise with a short recovery between repetitions (i.e. 35 s), resulting in significant decreases in distance from the first sprint (Boukhris et al. 2020), it is possible that a potential ergogenic mechanism of ACTP may prevent such reductions.

The present study demonstrated the benefits of ACTP ingestion on PP after 5msRT. However, Foster et al. (2014) reported that the ingestion of 1.5 g of ACTP had no beneficial effects on PP following repeated sprint cycling performance in physically active men.

Several mechanisms could be related to these results. Females have a greater sensitivity to pain and a lower pain threshold than males (Templeton 2020). Some gender differences in pain perception are exhibited in the descending pain modulator system, the brain-derived neurotrophic factor, the corticospinal motor pathway (Gasparin et al. 2020), and the impact of sex hormones on these pathways (Templeton 2020). 5-HT is a crucial neurotransmitter involved in the central mechanism of action of ACTP, which reduces the perception of pain (Pickering et al. 2006; Sandrini et al. 2007). Moreover, the influence of estrogen on 5-HT synthesis and reuptake increases the effectiveness of top-down pain inhibition (Paredes et al. 2019), and with the association of the central analgesic mechanism of action on the endocannabinoid system and stimulation of serotonergic pain inhibitory descending pathways (Przybyła et al. 2021), ACTP could lead to a better reduction in pain perception in females.

Overall, studies examining the effects of acute ACTP ingestion on repeated sprint performance included physically active participants (Foster et al. 2014, Delextrat et al. 2015, Park et al. 2016, Morgan et al. 2018), whereas the present study included well-trained participants, and athletes have a higher pain tolerance than non-athletes (Pettersen et al. 2020). Therefore, ACTP may be more likely to be ergogenic in this study.

This study indicated that ACTP ingestion improved FI (21.44%) and RPE compared with PLA. However, Morgan et al. (2019) showed that acute ingestion of ACTP was not effective in reducing RPE, increasing muscle activation or improving intramuscular disruption during fatiguing exercise in men. Several factors may explain the sex differences in RPE and fatigue for responses to ACTP during intermittent fatiguing exercise. It is well known that women are more resistant to fatigue than men, as they feel less peripheral fatigue (Wüst et al. 2008, Gentil et al. 2017). These sex differences are related to the difference in fiber type and composition (Toft et al. 2003), the oxidative system (Russ and Kent-Braun 2003), the level of glycolytic metabolism (Russ et al. 2005), sex hormones (New et al. 2000), the metabolic vasodilators of the muscle (Clifford and Hellsten 2004), and the level of sympathetic activation (Ettinger et al. 1996). Consequently, in this study, it is conceivable that ACTP, serving as a potential central regulator and decreasing strictness (Foster et al. 2014), may account for the observed enhancements in FI and RPE during the 5mSRT.

Considering that 5mSRT induces muscle damage, it leads to an increase in DOMS and a decrease in PRS in males (Boukhris et al. 2020). However, females might encounter lower levels of exercise-induced muscle damage than males, as estrogen plays a protective role in preserving muscle function (Minahan et al. 2015; Morawetz et al. 2020). The ACTP in this study reduced DOMS and increased PRS, which may be attributed to paracetamol’s mild anti-inflammatory properties (Koelsch et al. 2010; Graham et al. 2013). Thus, it could mitigate acute muscle damage, alleviate DOMS, and expedite muscle function recovery after 5mSRT.

Participants in our study were in different phases of the menstrual cycle (eight in the luteal phase and six in the follicular phase). This hormonal fluctuation can amplify the variability of the threshold and the perception of pain (Hellström and Anderberg 2003; da Silva et al. 2021). A previous study found that women’s strength levels differ depending on the phase of the menstrual cycle, which reaches its maximum in the middle of the cycle when estrogen levels are high (Sarwar 1996). Additionally, one study reported faster recovery of exercise-induced muscle lesions in the follicular phase (high estrogen concentration) of the menstrual cycle compared to women in the luteal phase (low estrogen concentration) (Markofski and Braun 2014). Exercise-induced muscle damage for DOMS and strength decline are influenced by hormonal fluctuations throughout the phases of the menstrual cycle, such as estrogen concentrations (Romero-Parra et al. 2021). Furthermore, the sensitivity of experimental pain varies during the menstrual cycle (Kowalczyk et al. 2010); however, fatigability and strength do not demonstrate such variability (Hunter 2014). Furthermore, the results show that the pharmacokinetics of ACTP differ between men and women. The maximum plasma concentration is higher and the half-life of ACTP is longer in women in both phases (follicular and luteal phase) than in men (Wójcicki et al. 1979).

Our study has some limitations: first, we did not take into account the effect of the individual phases of the menstrual cycle in the part of the results. Second, we did not evaluate muscle damage biomarkers such as creatine kinase (CK), lactate dehydrogenase (LDH), aspartate aminotransferase (ASAT) and alanine aminotransferase (ALAT), nor inflammation biomarkers (such as C-reactive protein CRP).

Future studies may consider additional measurements of biological markers of muscle damage after exercise and for 48 h after exercise, as pain and muscle damage have been reported hours and days after fatiguing exercise (Place et al. 2015).

Acute ingestion of ACTP at therapeutic doses is not associated with a health risk. However, chronic or toxic doses of ACTP significantly increase the risk of hepatic, renal, and gastrointestinal damage (Makin and Williams 1997).

Although hepatotoxicity generally occurs with doses of 10 g or more—well above the doses required to achieve an ergogenic effect (Wong and Graudins 2017), acute ingestion of 1.5 g of ACTP has shown no signs of hepatotoxicity, nor of drug accumulation, in healthy individuals or even in patients with severe liver disease (Forrest et al. 1979; Alchin et al. 2022). Furthermore, the pharmacokinetics of ACTP are not affected by exercise (Sawrymowicz 1997). When administered at recommended doses, ACTP is generally considered the safest and most effective analgesic available globally (Organization 2019). Currently, the use of ACTP is authorized by the World Anti-Doping Agency and is not listed among prohibited substances, although some have suggested that it should be included in the category of substances with therapeutic use exemptions (Lippi and Sanchis-Gomar 2014). The ethical aspects of using over-the-counter analgesics in athletes also need to be considered, as athletes who take such drugs, including NSAIDs, to reduce pain perception and enhance sports performance may in fact use them inappropriately (Alaranta et al. 2006). It is, therefore, crucial to make athletes aware of the potential risks associated with over-the-counter analgesics and to guide them towards using the safest option—namely, paracetamol—administered in the correct dosage and manner (specifically, an acute dose not exceeding 1.5 g and not for chronic use). A careful analysis of these aspects is essential before endorsing ACTP as an ergogenic aid.

## Conclusions

The present study showed the benefits of acute ingestion of ACTP on repeated high intensity short-term maximal performance and cognitive and mood status in well-trained women, with decreased pain perception, reduced perceived exertion, reduced delayed muscle pain, and improved perceived recovery. From a practical standpoint, administering acute, nontoxic doses to athletes prior to competition can improve physical and cognitive performances and the sensation of recovery and mood as well as reduce the perception of fatigue, muscle damage and pain.

## Data Availability

The data supporting the conclusions of this article can be made available by the authors, upon request.

## References

[CR1] Alaranta A, Alaranta H, Heliövaara M, Airaksinen M, Helenius I (2006) Ample use of physician-prescribed medications in Finnish elite athletes. Int J Sports Med 27:919–925 16586342 10.1055/s-2006-923811

[CR2] Alchin J, Dhar A, Siddiqui K, Christo PJ (2022) Why paracetamol (acetaminophen) is a suitable first choice for treating mild to moderate acute pain in adults with liver, kidney or cardiovascular disease, gastrointestinal disorders, asthma, or who are older. J Curr Med Res Opin 38(5):811–825 10.1080/03007995.2022.2049551 35253560

[CR3] Anderson BJ (2008) Paracetamol (acetaminophen): mechanisms of action. Paediatr Anaesth 18(10):915–921 18811827 10.1111/j.1460-9592.2008.02764.x

[CR4] Andersson DA, Gentry C, Alenmyr L, Killander D, Lewis SE, Andersson A, Bucher B, Galzi JL, Sterner O, Bevan S (2011) TRPA1 mediates spinal antinociception induced by acetaminophen and the cannabinoid Δ9-tetrahydrocannabiorcol. Nat Commun 2(1):1–11 10.1038/ncomms1559 22109525

[CR5] Bartley EJ, Fillingim RB (2013) Sex differences in pain a brief review of clinical and experimental findings. Br J Anaesth 111(1):52–58 23794645 10.1093/bja/aet127 PMC3690315

[CR6] Baur H, Hirschmüller A, Müller S, Gollhofer A, Mayer F (2007) Muscular activity in treadmill and overground running. Isokinet Exerc Sci 15(3):165–171

[CR7] Beal DJ, Weiss HM, Barros E, MacDermid SM (2005) An episodic process model of affective influences on performance. J Appl Psychol 90(6):1054–1068 16316265 10.1037/0021-9010.90.6.1054

[CR8] Bertolini A, Ferrari A, Ottani A, Guerzoni S, Tacchi R, Leone S (2006) Paracetamol new vistas of an old drug. CNS Drug Rev 12(3–4):250–275 17227290 10.1111/j.1527-3458.2006.00250.x PMC6506194

[CR9] Blecharz-Klin K, Piechal A, Pyrzanowska J, Joniec-Maciejak I, Kiliszek P, Widy-Tyszkiewicz E (2013) Paracetamol the outcome on neurotransmission and spatial learning in rats. Behav Brain Res 253:157–164 23850354 10.1016/j.bbr.2013.07.008

[CR10] Boddington MK, Lambert MI, Gibson ASC, Noakes TD (2001) Reliability of a 5-m multiple shuttle test. J Sports Sci 19(3):223–228 11256826 10.1080/026404101750095394

[CR11] Boddington MK, Lambert MI, Waldeck MR (2004) Validity of a 5-meter multiple shuttle run test for assessing fitness of women field hockey players. J Strength Cond Res 18(1):97–100 14971976 10.1519/1533-4287(2004)018<0097:voamms>2.0.co;2

[CR12] Botting RM (2000) Mechanism of action of acetaminophen: is there a cyclooxygenase 3? Clin Infect Dis 31(Supplement 5):S202–S210 11113024 10.1086/317520

[CR13] Boukhris O, Trabelsi K, Abdessalem R, Hsouna H, Ammar A, Glenn JM, Bott N, Irandoust K, Taheri M, Turki M (2020) Effects of the 5-m shuttle run test on markers of muscle damage, inflammation, and fatigue in healthy male athletes. Int J Environ Res Public Health 17(12):4375 32570815 10.3390/ijerph17124375 PMC7344466

[CR14] Boukhris O, Zghal F, Trabelsi K, Hsouna H, Abdessalem R, Ammar A, Elloumi M, Colson SS, Chtourou H (2022) The etiology of neuromuscular fatigue induced by the 5-m shuttle run test in adult soccer players. Kinesiology 54(2):347–356

[CR15] Carrier M, Debois N (2003) Émotions précompétitives et performance chez des escrimeurs de haut niveau : étude comparative du vécu émotionnel des sportifs d’élite lors de leurs meilleures et moins bonnes prestations. Cahiers De l’INSEP 34(1):337–340

[CR16] Cathel AM, Reyes BA, Wang Q, Palma J, Mackie K, Van Bockstaele EJ, Kirby LG (2014) Cannabinoid modulation of alpha2 adrenergic receptor function in rodent medial prefrontal cortex. Eur J Neurosci 40(8):3202–3214 25131562 10.1111/ejn.12690 PMC4205194

[CR17] Cayrou S, Dolbeault DP (2003) Version française du profile of mood states (POMS-f). J Behav Cogn Ther 13(2):83–88

[CR18] Chandrasekharan N, Dai H, Roos KLT, Evanson NK, Tomsik J, Elton TS, Simmons DL (2002) COX-3, a cyclooxygenase-1 variant inhibited by acetaminophen and other analgesic/antipyretic drugs: cloning, structure, and expression. Proc Natl Acad Sci 99(21):13926–13931 12242329 10.1073/pnas.162468699 PMC129799

[CR19] Clifford PS, Hellsten Y (2004) Vasodilatory mechanisms in contracting skeletal muscle. J Appl Physiol 97(1):393–403 15220322 10.1152/japplphysiol.00179.2004

[CR20] Cook DB, O’Connor PJ, Eubanks SA, Smith JC, Lee M (1997) Naturally occurring muscle pain during exercise assessment and experimental evidence. Med Sci Sports Exerc 29(8):999–1012 9268956 10.1097/00005768-199708000-00004

[CR21] Costello JT, Bieuzen F, Bleakley CM (2014) Where are all the female participants in Sports and Exercise Medicine research. Eur J Sport Sci 14(8):847–851 24766579 10.1080/17461391.2014.911354

[CR22] da Silva W, Machado ÁS, Lemos AL, de Andrade CF, Priego-Quesada JI, Carpes FP (2021) Relationship between exercise-induced muscle soreness, pain thresholds, and skin temperature in men and women. J Therm Biol 100:103051 34503798 10.1016/j.jtherbio.2021.103051

[CR23] Damien J, Mendrek A (2017) Santé mentale et douleur les différences hommes–femmes. Douleur analg, pp 1–8

[CR24] Dao T (2012) Sex differences in pain. J Am Dent Assoc 143(7):764–765 22751978 10.14219/jada.archive.2012.0264

[CR25] Delextrat A, O’Connor Ellis M, Baker CE, Matthew D, Sum A, Hayes LD (2015) Acetaminophen ingestion improves repeated sprint cycling performance in females: a randomized crossover trial. Kinesiology 47(2):145–150

[CR26] DeWall CN, MacDonald G, Webster GD, Masten CL, Baumeister RF, Powell C, Combs D, Schurtz DR, Stillman TF, Tice DM (2010) Acetaminophen reduces social pain behavioral and neural evidence. Psychol Sci 21(7):931–937 20548058 10.1177/0956797610374741

[CR27] Domes G, Schulze L, Böttger M, Grossmann A, Hauenstein K, Wirtz PH, Heinrichs H, Herpertz SC (2010) The neural correlates of sex differences in emotional reactivity and emotion regulation. Hum Brain Mapp 31(5):758–769 19957268 10.1002/hbm.20903 PMC6871188

[CR28] Eryılmaz SK, Aslankeser Z, Özdemir Ç, Özgünen K, Kurdak S (2019) The effect of 30-m repeated sprint exercise on muscle damage indicators, serum insulin-like growth factor-Iand cortisol. Biomed Hum Kinet 11(1):151–157

[CR29] Esh CJ, Mauger AR, Palfreeman RA, Al-Janubi H, Taylor L (2017) Acetaminophen (paracetamol) use beyond pain management and dose variability. Front Physiol 8:1092 29312002 10.3389/fphys.2017.01092 PMC5744234

[CR30] Ettinger SM, Silber DH, Collins BG, Gray KS, Sutliff G, Whisler SK, McClain JM, Smith MB, Yang Sinoway LI (1996) Influences of gender on sympathetic nerve responses to static exercise. J Appl Physiol 80(1):245–251 8847310 10.1152/jappl.1996.80.1.245

[CR31] Faul F, Erdfelder E, Lang AG, Buchner A (2007) G*Power 3: a flexible statistical power analysis program for the social, behavioral, and biomedical sciences. Behav Res Methods Comput 39(2):175–191 10.3758/bf03193146 17695343

[CR32] Fernandez Fernandez J, Zimek R, Wiewelhove T, Ferrauti A (2012) High-intensity interval training vs repeated-sprint training in tennis. J Strength Cond 26(1):53–62 10.1519/JSC.0b013e318220b4ff 21904233

[CR33] Forrest JA, Adriaenssens P, Finlayson N, Prescott L (1979) Paracetamol metabolism in chronic liver disease. Eur J Clin Pharmacol 15:427–431 499292 10.1007/BF00561743

[CR34] Foster JL, Taylor B, Chrismas C, Watkins SL, Mauger AR (2014) The influence of acetaminophen on repeated sprint cycling performance. Eur J Appl Physiol 114(1):41–48 24122176 10.1007/s00421-013-2746-0

[CR35] Gasparin A, Zortea M, Dos Santos VS, Carvalho F, Torres IL, de Souza A, Fregni F, Caumo W (2020) Brain-derived neurotrophic factor modulates the effect of sex on the descending pain modulatory system in healthy volunteers. Pain Med 21(10):2271–2279 32167540 10.1093/pm/pnaa027

[CR36] Gaudreau P, Nicholls A, Levy AR (2010) The ups and downs of coping and sport achievement: an episodic process analysis of within-person associations. J Sport Exerc Psychol 32(3):298–311 20587819 10.1123/jsep.32.3.298

[CR37] Gentil P, Campos MH, Soares S, Costa GDCT, Paoli A, Bianco A, Bottaro M (2017) Comparison of elbow flexor isokinetic peak torque and fatigue index between men and women of different training level. Eur J Transl Myol 27(4):7070 29299219 10.4081/ejtm.2017.7070 PMC5745383

[CR38] Graham GG, Davies MJ, Day RO, Mohamudally A, Scott KF (2013) The modern pharmacology of paracetamol: therapeutic actions, mechanism of action, metabolism, toxicity and recent pharmacological findings. Inflammopharmacology 21(3):201–232 23719833 10.1007/s10787-013-0172-x

[CR39] Gressier F, Calati R, Serretti A (2016) 5-HTTLPR and gender differences in affective disorders a systematic review. J Affect Disord 190:193–207 26519640 10.1016/j.jad.2015.09.027

[CR40] Grgic J (2022) What is the effect of paracetamol (acetaminophen) ingestion on exercise performance? Current findings and future research directions. Sports Med 52(3):431–439 35038139 10.1007/s40279-021-01633-4

[CR41] Grgic J, Mikulic P (2021) Effects of paracetamol (acetaminophen) ingestion on endurance performance: a systematic review and meta-analysis. Sports 9(9):126 34564331 10.3390/sports9090126 PMC8471630

[CR110] Haddad M, Chaouachi A, Castagna C, Hue O, Wong DP, Tabben M, Behm, DG, Chamari, K (2013) Validity and psychometric evaluation of the French version of RPE scale in young fit males when monitoring training loads. Sci Sports 28(2), e29-e35.

[CR42] Hatta T, Yoshizaki K, Ito Y, Mase M, Kabasawa H (2012) Reliability and validity of the digit cancellation test, a brief screen of attention. Psychologia 55(4):246–256

[CR43] Hawker GA, Mian S, Kendzerska T, French M (2011) Measures of adult pain: visual analog scale for pain (vas pain), numeric rating scale for pain (nrs pain), mcgill pain questionnaire (mpq), short-form mcgill pain questionnaire (sf-mpq), chronic pain grade scale (cpgs), short form-36 bodily pain scale (sf-36 bps), and measure of intermittent and constant osteoarthritis pain (icoap). Arthritis Care Res 63(S11):S240–S252 10.1002/acr.20543 22588748

[CR44] Hayes LD, Bickerstaff GF, Baker JS (2010) Interactions of cortisol, testosterone, and resistance training influence of circadian rhythms. Chronobiol Int 27(4):675–705 20560706 10.3109/07420521003778773

[CR45] Hellström B, Anderberg UM (2003) Pain perception across the menstrual cycle phases in women with chronic pain. Percept Mot Skills 96(1):201–211 12705527 10.2466/pms.2003.96.1.201

[CR46] Hunter SK (2014) Sex differences in human fatigability mechanisms and insight to physiological responses. Acta Physiol 210(4):768–789 10.1111/apha.12234 PMC4111134 24433272

[CR47] Ishida T, Sato T, Irifune M, Ki T, Nakamura N, Nishikawa T (2007) Effect of acetaminophen, a cyclooxygenase inhibitor, on Morris water maze task performance in mice. J Psychopharmacol 21(7):757–776 17606472 10.1177/0269881107076369

[CR48] Jones A, Zachariae R, Arendt-Nielsen L (2003) Dispositional anxiety and the experience of pain: gender-specific effects. Eur J Pain 7(5):387–395 12935790 10.1016/S1090-3801(02)00139-8

[CR49] Koelsch M, Mallak R, Graham GG, Kajer T, Milligan MK, Nguyen LQ, Newsham DW, Keh JS, Kettle AJ, Scott KF (2010) Acetaminophen (paracetamol) inhibits myeloperoxidase-catalyzed oxidant production and biological damage at therapeutically achievable concentrations. Biochem Pharmacol 79(8):1156–1164 19968966 10.1016/j.bcp.2009.11.024

[CR50] Kowalczyk WJ, Sullivan MA, Evans SM, Bisaga AM, Vosburg SK, Comer SD (2010) Sex differences and hormonal influences on response to mechanical pressure pain in humans. J Pain 11(4):330–342 19853526 10.1016/j.jpain.2009.08.004 PMC6174694

[CR51] Laurent CM, Green JM, Bishop PA, Sjökvist J, Schumacker RE, Richardson MT, Curtner-Smith M (2011) A practical approach to monitoring recovery development of a perceived recovery status scale. J Strength Cond 25(3):620–628 10.1519/JSC.0b013e3181c69ec6 20581704

[CR52] Lippi G, Sanchis-Gomar F (2014) Acetaminophen and sport performance: doping or what? Eur J Appl Physiol 114(4):881–882 24563094 10.1007/s00421-014-2852-7

[CR53] Lundberg TR, Howatson G (2018) Analgesic and anti-inflammatory drugs in sports Implications for exercise performance and training adaptations. Scand J Med Sci Sports 28(11):2252–2262 30102811 10.1111/sms.13275

[CR54] Maharaj H, Maharaj DS, Saravanan KS, Mohanakumar KP, Daya S (2004) Aspirin curtails the acetaminophen-induced rise in brain norepinephrine levels. Metab Brain Dis 19(1):71–77 15214507 10.1023/b:mebr.0000027418.33772.8b

[CR55] Makin AJ, Williams R (1997) Acetaminophen-induced hepatotoxicity predisposing factors and treatments. Adv Intern Med 42:453–483 9048127

[CR56] Manna SS, Umathe SN (2015) Paracetamol potentiates the antidepressant-like and anticompulsive-like effects of fluoxetine. Behav Pharmacol 26(3):268–281 25340977 10.1097/FBP.0000000000000104

[CR57] Markofski MM, Braun WA (2014) Influence of menstrual cycle on indices of contraction-induced muscle damage. J Strength Cond Res 28(9):2649–2656 24552791 10.1519/JSC.0000000000000429

[CR58] Mauger AR (2009) Anticipatory, feedforward and central regulation of pacing strategies in time trial cycling

[CR59] Mauger AR, Hopker JG (2013) The effect of acetaminophen ingestion on cortico-spinal excitability. Can J Physiol Pharmacol 91(2):187–189 23458204 10.1139/cjpp-2012-0213

[CR60] Mauger AR, Jones AM, Williams CA (2010) Influence of acetaminophen on performance during time trial cycling. J Appl Physiol 108(1):98–104 19910336 10.1152/japplphysiol.00761.2009

[CR61] McNair DM, Lorr M, Droppleman LF (1971) Manual profile of mood states

[CR62] Meltzer HY (1990) Role of serotonin in depression. Ann N Y Acad Sci 600:486–499 (**discussion 499–500**) 2252328 10.1111/j.1749-6632.1990.tb16904.x

[CR63] Mendiguren A, Pineda J (2004) Cannabinoids enhance *N*-methyl-d-aspartate-induced excitation of locus coeruleus neurons by CB1 receptors in rat brain slices. Neurosci Lett 363(1):1–5 15157983 10.1016/j.neulet.2004.02.073

[CR64] Merino Fernández M, Dal Bello F, Brabec Mota Barreto L, Brito CJ, Miarka B, López Díaz de Durana A (2019) State-trait anxiety and reduced emotional intelligence in combat sport athletes of different genders and competitive levels

[CR65] Minahan C, Joyce S, Bulmer AC, Cronin N, Sabapathy S (2015) The influence of estradiol on muscle damage and leg strength after intense eccentric exercise. Eur J Appl Physiol 115(7):1493–1500 25694209 10.1007/s00421-015-3133-9

[CR66] Morawetz D, Blank C, Koller A, Arvandi M, Siebert U, Schobersberger W (2020) Sex-related differences after a single bout of maximal eccentric exercise in response to acute effects: a systematic review and meta-analysis. J Strength Cond Res 34(9):2697–2707 30908366 10.1519/JSC.0000000000002867

[CR67] Morgan PT, Bowtell JL, Vanhatalo A, Jones AM, Bailey SJ (2018) Acute acetaminophen ingestion improves performance and muscle activation during maximal intermittent knee extensor exercise. Eur J Appl Physiol 118(3):595–605 29332237 10.1007/s00421-017-3794-7 PMC5805811

[CR68] Morgan PT, Bailey SJ, Banks RA, Fulford J, Vanhatalo A, Jones AM (2019) Contralateral fatigue during severe-intensity single-leg exercise: influence of acute acetaminophen ingestion. Am J Physiol Regul Integr Comp Physiol 317(2):R346–R354 31141387 10.1152/ajpregu.00084.2019 PMC6732432

[CR69] Naugle KM, Naugle KE, Fillingim RB, Riley JL 3rd (2014) Isometric exercise as a test of pain modulation effects of experimental pain test, psychological variables, and sex. Pain Med 15(4):692–701 24308352 10.1111/pme.12312 PMC4056589

[CR70] New G, Duffy SJ, Harper RW, Meredith (2000) Long-term oestrogen therapy is associated with improved endothelium-dependent vasodilation in the forearm resistance circulation of biological males. Clin Exp Pharmacol Physiol 27(1–2):25–33 10696525 10.1046/j.1440-1681.2000.03195.x

[CR71] O’connor P (1992) Psychological aspects of endurance performance. Endurance in Sport 139:145

[CR72] Oh SJ, Lee N, Nam KR, Kang KJ, Lee KC, Lee YJ, Seok JH, Choi JY (2023) Effect of developmental stress on the in vivo neuronal circuits related to excitation inhibition balance and mood in adulthood. Front Psychiatry 14:1086370 36846229 10.3389/fpsyt.2023.1086370 PMC9950095

[CR73] Ohashi N, Kohno T (2020) Analgesic effect of acetaminophen a review of known and novel mechanisms of action. Front Pharmacol 11:1916 10.3389/fphar.2020.580289 PMC7734311 33328986

[CR74] Organization WH (2019) WHO model list of essential medicines. WHO

[CR75] Organization WH (2022) World mental health report: transforming mental health for all. WHO

[CR76] Paredes S, Cantillo S, Candido KD, Knezevic NN (2019) An association of serotonin with pain disorders and its modulation by estrogens. Int J Mol Sci 20(22):5729 31731606 10.3390/ijms20225729 PMC6888666

[CR77] Park LL, Baker CE, Sum A, Hayes LD (2016) The influence of acetaminophen on sprint interval treadmill running: a randomized crossover trial. Kinesiology 48(1):58–62

[CR78] Pearcey GE, Bradbury-Squires DJ, Monks M, Philpott D, Power KE, Button DC (2016) Arm-cycling sprints induce neuromuscular fatigue of the elbow flexors and alter corticospinal excitability of the biceps brachii. Appl Physiol Nutr Metab 41(2):199–209 26799694 10.1139/apnm-2015-0438

[CR79] Pendleton M (1997) Reliability and validity of the Welsh rugby union shuttle run test. Unpublished BSc Dissertation, University of Wales Institute Cardiff, Cardiff, Wales, UK

[CR80] PettersenSD APM, Pettersen SA (2020) Pain processing in elite and high-level athletes compared to non-athletes. Front Psychol 11:1908 32849117 10.3389/fpsyg.2020.01908 PMC7399202

[CR81] Pickering G, Loriot MA, Libert F, Eschalier A, Beaune P, Dubray C (2006) Analgesic effect of acetaminophen in humans: first evidence of a central serotonergic mechanism. Clin Pharmacol Ther 79(4):371–378 16580905 10.1016/j.clpt.2005.12.307

[CR82] Pickering G, Macian N, Dubray C, Pereira B (2016) Paracetamol sharpens reflection and spatial memory: a double-blind randomized controlled study in healthy volunteers. Drug Design, Drug Des Devel Ther 10:3969 27980393 10.2147/DDDT.S111590 PMC5147402

[CR83] Place N, Ivarsson N, Venckunas T, Neyroud D, Brazaitis M, Cheng AJ, Ochala J, Kamandulis S, Girard S, Volungevičius G, Paužas H, Mekideche A, Kayser B, Martinez-Redondo V, Ruas JL, Bruton J, Truffert A, Lanner JT, Skurvydas A, Westerblad H (2015) Ryanodine receptor fragmentation and sarcoplasmic reticulum Ca2+ leak after one session of high-intensity interval exercise. Proc Natl Acad Sci U S A 112(50):15492–15497 26575622 10.1073/pnas.1507176112 PMC4687604

[CR84] Prior MJ, Lavins BJ, Cooper K (2012) A randomized, placebo-controlled trial of acetaminophen extended release for treatment of post-marathon muscle soreness. Clin J Pain 28(3):204–210 21760499 10.1097/AJP.0b013e318227cc4f

[CR85] Przybyła GW, Szychowski KA, Gmiński J (2021) Paracetamol—an old drug with new mechanisms of action. Clin Exp Pharmacol Physiol 48(1):3–19 32767405 10.1111/1440-1681.13392

[CR86] Rawls SM, Ding Z, Cowan A (2006) Role of TRPV1 and cannabinoid CB1 receptors in AM 404-evoked hypothermia in rats. Pharmacol Biochem Behav 83(4):508–516 16647109 10.1016/j.pbb.2006.03.011

[CR87] Redelinghuys C (2020) Serotonin/5-hydroxytryptamine (5-HT) physiology. South Afr J Anaesth Analg 26(6):S149-152

[CR88] Romero-Parra N, Cupeiro R, Alfaro-Magallanes VM, Rael B, Rubio-Arias J, Peinado AB, Benito PJ (2021) Exercise-induced muscle damage during the menstrual cycle: a systematic review and meta-analysis. J Strength Cond Res 35(2):549–561 33201156 10.1519/JSC.0000000000003878

[CR89] Russ DW, Kent-Braun JA (2003) Sex differences in human skeletal muscle fatigue are eliminated under ischemic conditions. J Appl Physiol 94(6):2414–2422 12562681 10.1152/japplphysiol.01145.2002

[CR90] Russ DW, Lanza IR, Rothman D, Kent-Braun JA (2005) Sex differences in glycolysis during brief, intense isometric contractions. Muscle Nerve 32(5):647–655 16025523 10.1002/mus.20396

[CR91] Sandrini M, Vitale G, Ruggieri V, Pini LA (2007) Effect of acute and repeated administration of paracetamol on opioidergic and serotonergic systems in rats. J Inflamm Res 56(4):139–142 10.1007/s00011-006-6113-z 17522810

[CR92] Sarwar R, Niclos BB, Rutherford OM (1996) Changes in muscle strength, relaxation rate and fatigability during the human menstrual cycle. J Physiol 493:267–272 8735711 10.1113/jphysiol.1996.sp021381 PMC1158967

[CR93] Sawrymowicz M (1997) The effect of exercise on the pharmacokinetics of acetaminophen and acetylsalicylic acid. Ann Acad Sci Fenn Math 9471923

[CR94] Sedıghı AR, Anbarıan M, Ghasemı MH (2019) Comparison of the electromyography activity of selected leg-dominant lower limb muscles during stance phase of running on treadmill and overground. TJSE 21(1):46–51

[CR95] Sood S, Howell J, Sundararajan V, Angus PW, Gow PJ (2013) Paracetamol overdose in Victoria remains a significant health-care burden. J Gastroenterol Hepatol 28(8):1356–1360 23489151 10.1111/jgh.12196

[CR96] Tang J, Gibson SJ (2005) A psychophysical evaluation of the relationship between trait anxiety, pain perception, and induced state anxiety. J Pain 6(9):612–619 16139780 10.1016/j.jpain.2005.03.009

[CR97] Tanner T, Aspley S, Munn A, Thomas T (2010) The pharmacokinetic profile of a novel fixed-dose combination tablet of ibuprofen and paracetamol. BMC Clin Pharmacol 10(1):10 20602760 10.1186/1472-6904-10-10 PMC2906415

[CR98] Templeton KJ (2020) Sex and gender issues in pain management. JBJS 102(Suppl 1):32–35 10.2106/JBJS.20.00237 32251123

[CR99] Toft I, Lindal S, Bønaa KH, Jenssen T (2003) Quantitative measurement of muscle fiber composition in a normal population. Muscle Nerve 28(1):101–108 12811780 10.1002/mus.10373

[CR100] Umathe SN, Manna SS, Utturwar KS, Jain NS (2009) Endocannabinoids mediate anxiolytic-like effect of acetaminophen via CB1 receptors. Prog Neuropsychopharmacol Biol Psychiatry 33(7):1191–1199 19580839 10.1016/j.pnpbp.2009.06.020

[CR101] Viberg H, Eriksson P, Gordh T, Fredriksson A (2014) Paracetamol (acetaminophen) administration during neonatal brain development affects cognitive function and alters its analgesic and anxiolytic response in adult male mice. Toxicol Sci 138(1):139–147 24361869 10.1093/toxsci/kft329

[CR102] Vincent K, Tracey I (2008) Hormones and their interaction with the pain experience. Pain Rev 2(2):20–24 10.1177/204946370800200206 PMC4589942 26526773

[CR103] Wójcicki J, Gawrońska-Szklarz B, Kazimierczyk J, Baskiewicz Z, Raczyński A (1979) Comparative pharmacokinetics of paracetamol in men and women considering follicular and luteal phases. Arzneimittelforschung 29(2):350–352 582149

[CR104] Wong A, Graudins A (2017) Risk prediction of hepatotoxicity in paracetamol poisoning. Clin Toxicol 55(8):879–892 10.1080/15563650.2017.1317349 28447858

[CR105] Wüst RC, Morse CI, De Haan A, Jones DA, Degens H (2008) Sex differences in contractile properties and fatigue resistance of human skeletal muscle. Exp Physiol 93(7):843–850 18296492 10.1113/expphysiol.2007.041764

